# Altered somatosensory neurovascular response in patients with Becker muscular dystrophy

**DOI:** 10.1002/brb3.985

**Published:** 2018-04-24

**Authors:** Ulrich Lindberg, Christina Kruuse, Nanna Witting, Stine Lundgaard Jørgensen, John Vissing, Egill Rostrup, Henrik Bo Wiberg Larsson

**Affiliations:** ^1^ Functional Imaging Unit Department of Clinical Physiology Nuclear Medicine and PET Rigshospitalet Glostrup University of Copenhagen Glostrup Denmark; ^2^ Lundbeck Foundation Center for Neurovascular signalling (LUCENS) Rigshospitalet Glostrup University of Copenhagen Glostrup Denmark; ^3^ Neurovascular Research Unit Department of Neurology Herlev Gentofte Hospital University of Copenhagen Herlev Denmark; ^4^ Copenhagen Neuromuscular Center Department of Neurology Rigshospitalet University of Copenhagen Denmark

**Keywords:** Becker muscular dystrophy, BOLD signal, case–control study, neurovascular coupling, somatosensory evoked potentials

## Abstract

**Introduction:**

Patients with dystrophinopathies show low levels of neuronal nitric oxide synthase (nNOS), due to reduced or absent dystrophin expression, as nNOS is attached to the dystrophin‐associated protein complex. Deficient nNOS function leads to functional ischemia during muscle activity. Dystrophin‐like proteins with nNOS attached have also been identified in the brain. This suggests that a mechanism of cerebral functional ischemia with attenuation of normal activation‐related vascular response may cause changes in brain function.

**Methods:**

The aim of this study was to investigate whether the brain response of patients with Becker muscular dystrophy (BMD) is dysfunctional compared to that of healthy controls. To investigate a potential change in brain activation response in patients with BMD, median nerve somatosensory evoked stimulation, with stimulation durations of 2, 4, and 10 s, was performed while recording electroencephalography and blood oxygen level‐dependent (BOLD) functional magnetic resonance imaging.

**Results:**

Results in 14 male patients with BMD (36.2 ± 9.9 years) were compared with those of 10 healthy controls (34.4 ± 10.9 years). Compared to controls, the patients with BMD showed sustained cortical electrical activity and a significant smaller BOLD activation in contralateral primary somatosensory cortex and bilaterally in secondary somatosensory cortex. In addition, significant activation differences were found after long duration (10 s) stimuli in thalamus.

**Conclusion:**

An altered neurovascular response in patients with BMD may increase our understanding of neurovascular coupling and the pathogenesis related to dystrophinopathy and nNOS.

## 1 INTRODUCTION

Duchenne (DMD) and Becker (BMD) muscular dystrophies, collectively called dystrophinopathies, are *X*‐linked and caused by mutations in the dystrophin gene. DMD and BMD are characterized by progressive muscle wasting, cardiomyopathy, and a reduced life expectancy (Bushby et al., 1993, 2010a,b; Emery, 2002). However, a slower progression and less severe phenotype are seen in patients with BMD compared to DMD. Patients with BMD, unlike patients with DMD, typically carry in‐frame mutations of the dystrophin gene preserving some residual dystrophin function (Anthony et al., 2011). In addition to muscle and cardiac involvement, dystrophinopathies also involve cognitive symptoms. Patients with DMD score one standard deviation below normal in IQ tests (Cotton, Voudouris, & Greenwood, 2001), and behavioral and psychiatric abnormalities have also been reported (Haenggi & Fritschy, 2006; Waite, Brown, & Blake, 2012; Waite, Tinsley, Locke, & Blake, 2009; Young et al., 2007). Patients with BMD show similar deficits, though with less severity and more variability (Chamova et al., 2013; Melo et al., 1995). The pathophysiology of brain involvement in dystrophinopathies is not yet understood.

Reduced or absent dystrophin expression causes the membrane‐bound dystrophin‐associated glycoprotein complex (DGC), present in skeletal and cardiac muscle cells (Hoffman & Kunkel, 1989; Matsumura & Campbell, 1994) as well as the neuromuscular junction and the brain (Nichols, Takeda, & Yokota, 2015) to be dysfunctional. This in turn may affect the regulation and distribution of brain blood flow due to NO's vasodilatory effect exerted on the smooth muscle cells of the brain arterioles (Attwell et al., 2010).

In patients with DMD, compared with matched controls, structural abnormalities such as smaller total brain volume, reduced cerebral gray matter volume, and perfusion deficits were recently reported (Doorenweerd et al., 2014, 2017). Functional connectivity studies showed an altered neurovascular activation in DMD patients with blood oxygen level‐dependent (BOLD) changes in the motor cortex measured by resting state functional magnetic resonance imaging (fMRI) (Lv et al., 2011). Such studies suggest that dystrophin or proteins in the DGC play an important functional role in the brain. Using positron emission tomography (PET), reduced brain tissue glucose metabolism in sensorimotor areas was reported in patients with DMD (Lee et al., 2002). Functional brain MRI studies in patients with DMD are few, and no studies have been undertaken with regard to BMD. Reports from a different muscular disease, myotonic dystrophy, show some evidence of abnormalities in the BOLD response, both resting state and task‐induced responses (Okkersen et al., 2017).

We hypothesized that a deficiency in dystrophin, most likely associated with a reduced nNOS expression in the brain similar to that shown in dystrophin‐deficient muscle, leads to an altered neurovascular response in patients with BMD. As a result, the neurovascular coupling would be altered in BMD patients compared with healthy subjects. An attenuation of NO production by reduced nNOS levels would cause decreased cerebrovascular responsiveness to neuronal stimulation causing a nonsufficient supply of oxygen and glucose, in particular during high demanding periods of repeated brain activation. In this study, we apply both somatosensory evoked potentials (SEP) and fMRI allowing a comparison of the neurovascular coupling with median nerve stimulation in the sensorimotor brain area.

## 2 MATERIALS AND METHODS

### 2.1 Participants

Fourteen males affected by BMD, carrying previously reported mutations in the dystrophin gene, were included (Witting et al., 2014). Ten healthy males with comparable handedness and age were studied for comparison (Table 1). The Ethics Committee of the Capital Region of Denmark approved the study, and all subjects gave written informed consent according to the Helsinki Declaration before participating. This study was part of a larger setup in which treatment with sildenafil was investigated and compared in the patients (Lindberg et al., 2017; Witting et al., 2014). All subjects underwent two identical somatosensory electrical stimulation tasks: one outside the scanner with recorded EEG and one inside the scanner during BOLD‐fMRI recording. All parameters were kept constant between the two runs.

**Table 1:** Demography and group statistical comparisons

	Becker patients *N* = 14	Healthy controls *N* = 10	*p*‐value
Age	36.2 ± 9.9 years	34.4 ± 10.9 years	.74^a^
Stimulation intensity	4.29 ± 1.07 mA	4.81 ± 1.07 mA	.26^b^
Handedness (right)	93%	90%	.85^b^
SEP N20‐P26 difference	0.89 ± 0.49 μV	1.29 ± 0.79 μV	.14^a^

Mean ± standard deviation.

a Two‐sample two‐sided *t* test.

b Mann–Whitney *U* test.

### 2.2 Electrical stimulation

**Figure 1:**
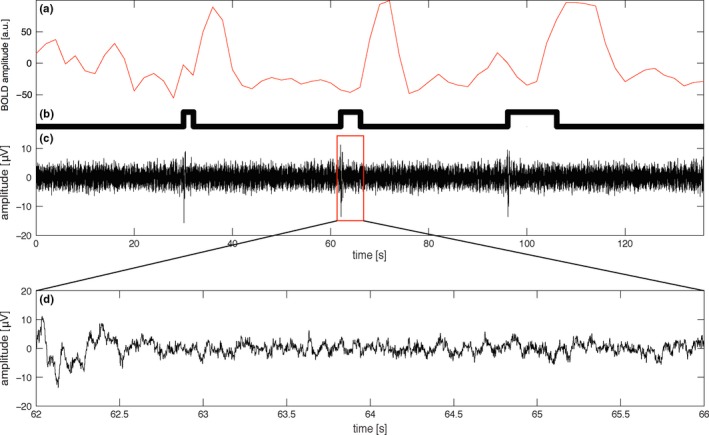
(Top) Mean time courses of the three different durations (2, 4, and 10 s) from one subject. (a) BOLD response. (b) Stimulation vector. (c) EEG response from the contralateral channel. (Bottom) (d) An amplified image of the EEG trace for the 4‐s stimulation period

The delivery of the electrical impulses was carried out using a DS7A & DS7AH high‐voltage constant current electrical stimulator (Digitimer Ltd, United Kingdom). Median nerve stimulation was performed on the dominant hand, as determined by the Edinburgh Handedness Scale (Oldfield, 1971). Each stimulus consisted of a brief square pulse of 200 μs at an intensity of 80% of the motor threshold. The stimulation paradigm consisted of three different stimulation block lengths (2, 4, and 10 s). All blocks were repeated 10 times in a random order with a stimulation frequency of 5 Hz and an interstimulus block interval of 30 s to allow the BOLD response to settle down (Figure 1). This adds to a total of 30 stimulation blocks with a total of 800 stimulations during the run.

### 2.3 Magnetic resonance imaging

Magnetic resonance imaging was performed on a 3.0T Philips Achieva scanner (Philips Medical Systems, Best, The Netherlands) using a 32‐channel head coil. Anatomical images were acquired using a MPRAGE three‐dimensional turbo field‐echo sequence (137 sagittal slices of 1.1 mm thickness; in‐plane resolution 1 × 1 mm^2^; TR 6,900 ms; TE 2.8 ms; TI 900 ms; and flip angle 9°).

Acquisition of the functional images was performed using an echo planar imaging sequence (24 axial slices of 4.0 mm thickness; slice gap 0.1 mm; field of view 230 × 230 mm^2^; in‐plane acquired resolution 1.8 × 1.8 mm^2^; TR 2,000 ms; TE 35 ms; flip angle 76°; with SENSE (SENSitivity Encoding) turned off; 545 volumes). The slices were angulated and aligned to the extremities of the anterior and posterior parts of corpus callosum. Magnetization saturation effects were avoided by discarding the first two volumes of each scan. An online general linear model was performed on the Philips scanner software to monitor the performance and to assure that the electrical stimulator was kept in place.

### 2.4 Somatosensory evoked potentials

The recording of the somatosensory evoked potentials (SEP) outside the scanner was done using a 32‐channel actiCap system (Brain Products GmbH, Munich, Germany) placed according to the international 10–20 standard electrode placement system. The participants were placed on the same table as used inside the MRI scanner and the SEP data from channels C3 and C4 were acquired at 5 kHz using the BrainVision Recorder software (Brain Amps GmbH, Germany).

In total, 800 electrical median nerve stimulations were acquired. For each subject, an average of all 800 somatosensory evoked potentials (SEP) from 1 to 200 ms was computed, followed by averaging the subjects within the two groups individually.

**Figure 2:**
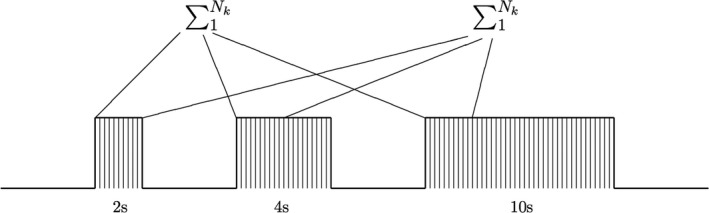
Boxcar stimulus setup (5 Hz). Ten of each block was presented in a randomized order. The narrow lines represent each electrical stimulus within each stimulation block

For all the stimulation blocks, each stimulation number in the train of stimulations, *s*_*n*_, was averaged individually to investigate the time effect of each stimulus in the train (Figure 2).

$$\bar{S_{n}}\left(t\right)=\frac{1}{N_{k}}\sum_{k=1}^{N_{k}}S_{n,k}\left(t\right)$$

where $\bar{S_{n}}$ is the mean signal of all the stimulations, *n* is the stimulation number in the block, and *k* is the repetition number and *N*_*k*_ is

$$N_{k}=\left\{\begin{array}{cc} 30 & \text{for}\;\;n\leq 10\\ 20 & \text{for}\;\;n>10\\ 10 & \text{for}\;\;n>20 \end{array}\right.\leq 20$$

The root mean square (RMS) value of each 200 ms stimulation period, $\bar{S_{n}}$, was then calculated and normalized to the value of the first stimulation. Group comparison was then performed using a two‐sample *t* test in MATLAB (The MathWorks, Natick, MA, USA) for each of the stimulations (*n* = 2–20).

### 2.5 Postprocessing

Postprocessing and statistical analysis of the MRI data were performed using the FEAT (fMRI Expert Analysis Tool) version 6.00 in the FSL software package (Smith et al., 2004) (FMRIB Software Library, Functional Magnetic Resonance Imaging of the Brain Center, Department of Clinical Neurology, University of Oxford, Oxford, UK). The data were brain extracted with a fractional intensity threshold of 0.3, motion corrected to the central volume using 6 degrees of freedom, high‐pass filtered at 1/90 s, and spatially smoothed using a full‐width half‐maximum Gaussian kernel of 5 mm before general linear modeling (GLM) was performed. Three regressors defining the stimulus onsets of the 2‐, 4‐, and 10‐s stimulation blocks (as explained in the section on electrical stimulation) were convolved with a double‐gamma hemodynamic response function and used in the GLM. Both activations and deactivations were investigated. Significant clusters were determined from the *Z*‐statistical images by a threshold of *Z* > 2.3 and a (corrected) cluster significance threshold of *p* = .05 (Jezzard, Matthews, & Smith, 2003). Familywise error correction (FWE) was applied. Functional images were registered to the structural T1 image using boundary‐based registration, and the normalization to MNI‐152 standard space was carried out between the structural T1 images and a 1 mm MNI‐152 standard brain by a linear (12 degrees of freedom) and nonlinear registration (warp resolution of 10 mm, 10 mm, 10 mm). Groupwise analysis was carried out using FLAME (FMRIB's Local Analysis of Mixed Effects) stage 1 with a column describing the patients and one describing the controls. Four contrasts were tested as follows: C1) average of the patient group, C2) average of the control group, C3) patients greater than controls, and C4) controls greater than patients. The group comparison results were overlaid on the T1 1 mm MNI‐152 standard brain, and anatomical locations were defined using the Juelich histological atlas (Eickhoff et al., 2005). To investigate effects of stimulus duration on BOLD amplitude, regression analysis testing the temporal effects, as well as group differences, was carried out on contralateral SI, SII, and thalamus based on Juelich histological atlas segmentations. The postprocessing of the SEP data included band‐pass filtering with cutoff at 1–500 Hz, segmentation of the individual stimuli, and averaging of these, all done in MATLAB.

## 3 RESULTS

Age and handedness were not statistically different between the two groups (Table 1). The intensity of the applied stimulus to the dominant median nerve was (mean ± *SD*) 4.29 ± 1.07 mA in the BMD group and 4.81 ± 1.07 mA in the control group, which was not significantly different when compared using a Mann–Whitney *U* test (*p* = .26).

**Figure 3:**
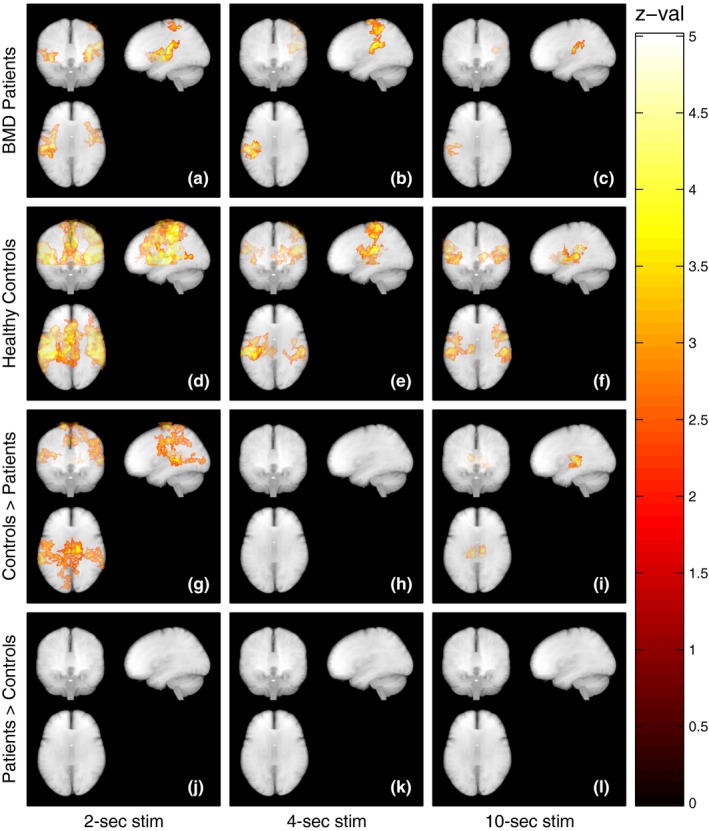
Mean BOLD activation in the BMD group (a–c). The 2‐s stimulation (a) gives a strong BOLD response in contralateral primary sensory cortex together with bilaterally in secondary somatosensory cortex. The response to 4‐s stimulation (b) covers the contralateral side both in primary‐ and secondary somatosensory cortex. The 10‐s stimulation (c) only activates the contralateral secondary somatosensory cortex. Mean BOLD activation in the control group (d–f). Both 2‐s stimulation (d) and 4‐s stimulation (e) elicit a strong BOLD response in contralateral primary sensory cortex in addition to bilaterally secondary somatosensory cortex and thalamus. 10‐s stimulation (f) shows activation only located to contralateral primary somatosensory cortex, bilateral secondary somatosensory cortex, and contralateral thalamus. Comparison between activation maps where controls have stronger activation than patients with BMD at 2‐ (g), 4‐ (h), and 10‐s (i) stimulation. Controls show a larger activation in the primary somatosensory cortex SI as well as the secondary somatosensory cortex SII to all stimulus durations. Thalamus differences are seen for the 10‐s stimulation periods. No significant areas were found testing for patients with BMD showing stronger activation than controls (j), (k), and (l)

The voxelwise group statistics are reported in Figure 3, with the list of significant clusters summarized in Table 2.

**Table 2:** Statistical information, both *p*‐value and *Z*‐score, of the significant cluster found in the voxelwise group comparison

Contrast	Index	Voxels	Anatomical location	*p*‐value	*Z*‐max	*Z*‐max *X* (mm)	*Z*‐max *Y* (mm)	*Z*‐max *Z* (mm)
2‐s stimulation								
BMD mean	3	13,800	Left insula, Left secondary somatosensory cortex	1.19e‐07	4.8	−38	−19	10
	2	7,372	Right insula, Right secondary somatosensory cortex	1.32e‐04	4.31	39	−1	−7
	1	3,104	Left primary somatosensory cortex	0.0418	3.83	−44	−41	61
CON mean	2	143,067	Bilateral primary somatosensory cortex, Bilateral secondary somatosensory cortex, Bilateral Insula	2.97e‐40	5.52	−56	−22	48
	1	38,289	Premotor cortex	4.63e‐16	4.52	−4	−20	47
BMD > CON	–	–	–	–	–	–	–	–
CON > BMD	6	10,624	Left primary somatosensory cortex	3.46e‐06	3.73	−57	−24	47
	5	9,925	Premotor cortex	7.27e‐06	3.82	−3	−20	47
	4	7,401	Right secondary somatosensory cortex	1.27e‐04	3.94	60	−37	12
	3	5,060	Premotor cortex	2.46e‐03	3.97	13	−17	77
	2	4,530	Left secondary somatosensory cortex	5.09e‐03	3.87	−59	−36	6
	1	3,690	Left visual cortex V1	0.0171	3.13	−20	−66	4
4‐s stimulation								
BMD mean	2	8,319	Left secondary somatosensory cortex	2.95e‐04	4.54	−53	−30	28
	1	6,656	Left primary somatosensory cortex	1.58e‐03	3.99	−45	−42	60
CON mean	3	19,963	Left secondary somatosensory cortex	1.96e‐08	4.47	−54	−28	19
	2	11,563	Left primary somatosensory cortex	1.50e‐05	4.19	−57	−21	49
	1	9,445	Right secondary somatosensory cortex	1.01e‐05	4.23	60	−29	17
BMD > CON	–	–	–	–	–	–	–	–
CON > BMD	–	–	–	–	–	–	–	–
10‐s stimulation								
BMD mean	1	3,119	Left secondary somatosensory cortex	0.0409	3.64	−41	−18	15
CON mean	3	16,579	Left thalamus, Left secondary somatosensory cortex	1.04e‐08	4.11	−17	−25	9
	2	10,237	Right insula	5.25e‐06	4.42	62	6	7
	1	7,940	Right secondary somatosensory cortex	6.77e‐05	4.39	57	−22	20
BMD > CON	–	–	–	–	–	–	–	–
CON > BMD	2	5,005	Left thalamus	2.65e‐03	3.84	−5	−27	−2
	1	3,239	Right thalamus	0.034	3.99	12	−18	12

BMD, Becker muscular dystrophy; CON, Healthy controls.

The voxel coordinates refer to the most significant voxel in each cluster based on the T1 1 mm MNI‐152 standard brain.

### 3.1 Blood oxygen level‐dependent response

The two‐second median nerve stimulation elicited a significant positive BOLD response in contralateral primary somatosensory cortex (SI) and bilateral activation in secondary somatosensory cortex (SII) in both groups (Figure 3a, patients and Figure 3d, controls). In addition, the control group showed activation in the supplementary motor area (SMA) to the median nerve stimulation. The group comparison analysis showed a significantly stronger activation in the control group, mainly localized bilaterally in the SII and contralateral SI as well as in the premotor cortex (Figure 3g). The BMD group had no areas of significantly stronger activations compared to the control group (Figure 3j).

The four‐second stimulation induced the same activation pattern to median nerve stimulation as the two‐second stimulation with the exception that activation of the ipsilateral SII was absent in the BMD group (Figure 3b, patients and Figure 3e, controls). Group comparison did not reveal differences in any areas (Figure 3h,k).

The group difference was further enhanced during the ten‐second stimulation, where the BMD group only showed activation in the contralateral SII (Figure 3c, patients and Figure 3f, controls). The activation map of the control group was similar during the range of stimulation durations, though with a decreased contralateral SI activation during the 10‐s stimulation. Comparing the two groups revealed bilateral differences in thalamus activation with controls showing stronger activation (Figure 3i). The BMD group showed no areas of stronger activation compared to the control group (Figure 3l).

Analysis of voxelwise deactivations showed no consistent pattern for any of the stimulation durations and no significant differences in the group comparison.

Regression analysis of the atlas‐based regions showed a significant decrease with stimulus duration in contralateral SI in both groups (*p* < .001), but with no group difference when comparing slopes. Neither the SII nor thalamus showed any significant decrease in BOLD amplitude or a significant group difference as the effect of stimulus duration.

### 3.2 Somatosensory Evoked Potentials

No significant difference (*p* = .14) was found comparing the N20‐P26 SEP amplitude of the patient and control groups.

**Figure 4:**
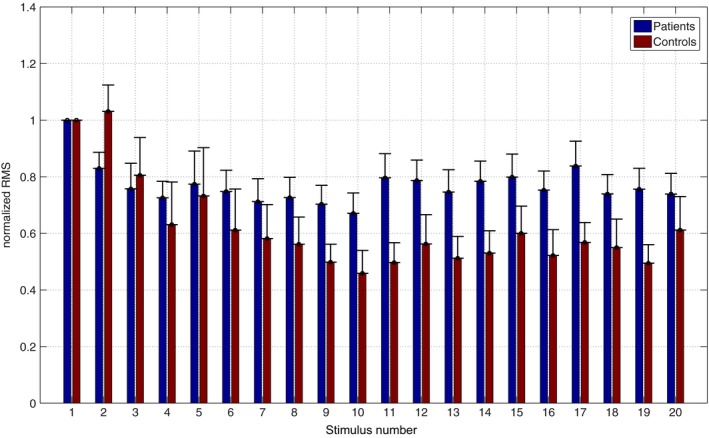
Normalized root mean square (RMS) value of neuronal response of the individual stimulations in a stimulus train. Patients have a more sustained activity with significant higher RMS after nine stimulations compared to healthy controls. Error bars are standard error of the mean

The RMS value of each stimulus number calculated from equation 1 showed that patients with BMD had a significantly (*p* < .02) increased power when compared to healthy controls from stimulus 9 and onwards (Figure 4).

## 4 DISCUSSION

Investigating the neurovascular response in patients with BMD, we found a decreased BOLD response contralateral to the stimulation in the primary somatosensory cortex (SI), as well as bilaterally in the secondary somatosensory cortex (SII), and thalamus, compared with healthy controls. In addition, a sustained EEG activity was detected during electrical somatosensory stimulation in patients with BMD in contrast to controls where the electrical activity dropped and levelled off, which may represent neural adaptation. Changes in the neurovascular response and functional stimulation‐based activations using MRI have not previously been reported in patients with BMD compared to healthy controls. Sensory function was chosen for investigation of neurovascular coupling in patients with BMD, as they have no clinically apparent deficit in this area and, for this reason, performance issues do not confound the results. The decrease in BOLD response in patients could be due to an insufficient neurovascular coupling, in particular involving the NO cascade which could be analogous to the reduced blood flow response during muscle activation reported in patients with BMD. This interpretation is supported by reports of decreased expression of nNOS in neurons that lack dystrophin (Sogos, Reali, Fanni, Curto, & Gremo, 2003) and corresponds well with the decrease in muscle nNOS found in our patient population (Witting et al., 2014). With dystrophin present in neurons and the microvasculature of the brain, a cerebral functional ischemia may exist similar to that of skeletal muscles in the patients with BMD (Rando, 2001), though with a less remarkable clinical impact. Insufficient NO in the microvasculature of skeletal muscle is known to increase the susceptibility to functional ischemia, which leads to muscle injury during prolonged hypoxic muscle strain (Martin et al., 2012; Thomas, 2013). In the present study of patients with BMD, treatment with a phosphodiesterase 5 (PDE5) inhibitor resulted in a significant increase in the BOLD signal in somatosensory and visual areas, which indicates that the NO‐cGMP cascade could be involved (Lindberg et al., 2017).

As previously reported in these patients, blood flow response in the brachial artery was reduced during exercise (Witting et al., 2014), though without improved response after treatment with sildenafil, in contrast to the current findings. The blood flow changes represent a large artery response, but are still in line with reports on dysfunctional blood flow in the dystrophinopathies. Baseline cerebral perfusion is regulated by both large artery and small artery responses, and influences the size of the BOLD response due to possible vascular ceiling effects (Birn & Bandettini, 2005; Buxton, Uludağ, Dubowitz, & Liu, 2004) which could affect brain activation BOLD data. A lower CBF has been reported in young DMD boys compared to healthy age‐matched controls (Doorenweerd et al., 2017), which indicates that the patients could suffer from a dysfunctional stimulus‐induced CBF regulation leading to a decreased BOLD response.

The neuronal output to the vascular system during the median nerve stimulation was increased in the BMD group (Figure 4), contrasting to a decrease observed in the BOLD response. This may very well be in line with findings from a study on somatosensory evoked potentials in patients with myotonic dystrophy, although a different pathophysiology, which showed abnormal cortical excitability to ongoing median nerve stimulation when compared to healthy controls (Mochizuki et al., 2001). In general, amplitude coupling between neural activity and vascular response is often considered nearly linear in the healthy brain (Li & Freeman, 2007; Logothetis, Pauls, Augath, Trinath, & Oeltermann, 2001), but a change in the coupling pattern was observed in the present study. A similar uncoupling of neuronal activity and metabolism has been reported in patients with mitochondrial encephalomyopathy, lactic acidosis, and stroke‐like episodes (MELAS) where episodes of a high paroxystic activity occur concurrently with a diminished metabolism and cerebral flow (Yeh et al., 2013).

A decreased activation was seen bilaterally in thalamus for the 4‐ and 10‐s stimulation periods in the patients with BMD vs. controls. The thalamus, in addition to passively relaying information to the cortex, appears to be involved in response magnitude, firing mode, and synchrony of neurons (Saalmann & Kastner, 2011). The changes in neuronal activation discovered in this study could suggest changes in magnitude modulation in BMD patients, with altered sensory inputs in the thalamus leading to an altered adaptation measured in the cortex. A thalamic involvement in higher order functions has previously been described (Saalmann & Kastner, 2011). Our results, however, suggest that the thalamic activation represents the stimulation of the somatosensory cortical areas and that this is merely a consequence of a general change in neurovascular coupling, leading to a lower BOLD response in thalamus.

Stimulation strength was kept constant between the two groups, and variability in stimulation intensity could therefore not explain the intergroup differences in the results. A previous study on cortical excitability in patients with DMD did not show any difference when compared to healthy controls (Yayla et al., 2008). In our study, the group comparison showed more prominent differences in the secondary somatosensory cortex in the control group compared to the patients with BMD (Figure 3). According to Backes et al. (Backes, Mess, van Kranen‐Mastenbroek, & Reulen, 2000), the BOLD response remains constant regardless of the applied stimulus intensity between 50 and 100% of motor threshold. Stimulus strength is therefore unlikely to have caused any group differences in the SII.

The current study showed a change in neurovascular response in BMD patients compared with controls, which could represent a change in the neurovascular coupling. A further investigation of the functional impact of the insufficient neurovascular coupling in patients with BMD needs to be established. Further studies are needed to determine whether a deficient neurovascular coupling could impact the cognitive function.

## CONFLICT OF INTEREST

None of the authors have any conflict to declare.
